# Differential Attachment of Engineered Oral Soft Tissues to Implant Surfaces

**DOI:** 10.3390/dj14030150

**Published:** 2026-03-06

**Authors:** Nour Jalaleddine, Emilia Barker, Kirsty Franklin, Mohamed Jamal, Momen A. Atieh, Zaid H. Baqain, Keyvan Moharamzadeh

**Affiliations:** 1 Hamdan Bin Mohammed College of Dental Medicine, Mohammed Bin Rashid University of Medicine and Health Sciences, Dubai Health, Dubai P.O. Box 505055, United Arab Emirates; nour.jalaleddine@dubaihealth.ae (N.J.); mohamed.jamal@dubaihealth.ae (M.J.); momen.atieh@dubaihealth.ae (M.A.A.); zaid.baqain@dubaihealth.ae (Z.H.B.); 2 School of Clinical Dentistry, University of Sheffield, Sheffield S10 2TA, UK; emilia.barker@sheffield.ac.uk (E.B.); k.l.franklin@sheffield.ac.uk (K.F.); 3 Sir John Walsh Research Institute, Faculty of Dentistry, University of Otago, Dunedin 9016, New Zealand; 4 School of Dentistry, University of Jordan, Amman 11942, Jordan

**Keywords:** implant, soft tissue attachment, oral mucosa, tissue engineering

## Abstract

**Background/Objectives**: The formation of a soft tissue seal through mucosal integration around dental implants is critical for potentially achieving long-term peri-implant health and clinical success. Understanding how different implant and abutment surfaces interact with individual layers of the oral mucosa remains limited. This study aimed to compare the differential attachment of tissue-engineered oral epithelium, connective tissue, and full-thickness human oral mucosa to various implant and abutment materials and surface topographies. **Methods**: Sand-blasted, large-grit, acid-etched (TiZr-SLA), machined TiZr (TiZr-M), machined zirconia (ZrO_2_-M), polished zirconia (ZrO_2_-P), and machined PEEK rods, along with commercially available titanium and ZrO_2_ healing abutments, were inserted into 3D oral mucosal models following a 4-mm punch biopsy. Inflammation was induced using Escherichia coli lipopolysaccharide. Analyses included histology, PrestoBlue viability assay, scanning electron microscopy, and ELISA quantification of cytokines IL-1β, IL-6, and IL-8. **Results**: Epithelial attachment was greater on TiZr-SLA, ZrO_2_-P, and PEEK-M (*p* < 0.05 and *p* < 0.01) surfaces compared with TiZr-M and ZrO_2_-M. TiZr-SLA exhibited the highest connective tissue attachment (*p* < 0.05). Commercial titanium and ZrO_2_ healing abutments demonstrated the highest post-pull PrestoBlue viability and overall soft tissue attachment. SEM confirmed cell retention on all implant surfaces. Elevated IL-1β levels were detected in models exposed to ZrO_2_-M and PEEK-M, whereas IL-6 and IL-8 levels were not influenced by any material or surface topography. **Conclusions**: In vitro epithelial and connective tissue responses are influenced by implant material, surface topography, and design. Rough TiZr-SLA surfaces promote superior connective tissue attachment, while smooth commercial abutments support optimal overall soft tissue integration. These findings highlight the importance of surface engineering in preclinical optimization of peri-implant soft tissue attachment.

## 1. Introduction

Mucosal integration to dental implant abutments has become a major focus of implant surface research in the past decade [1]. Soft tissue sealing and the barrier function provided by optimal gingival mucosal contact with an abutment are essential to protect the underlying bone and achieve long-term peri-implant tissue health and stability [2,3]. Commonly used cell culture systems for the investigation of implant-soft tissue attachment include monolayer (2D) cultures of human oral keratinocytes [4,5] and fibroblasts [6,7]. However, these culture systems do not reproduce the three-dimensional (3D) architecture of the peri-implant tissue anatomy, and the results may not be extrapolated to the clinical environment. Animal studies on implant-soft tissue attachment also have several limitations, including ethical issues, costs, technical challenges, and scalability [8]. A recent systematic review and meta-analysis of histological animal studies further indicated that available evidence is limited and inconclusive regarding the influence of transmucosal implant component characteristics on peri-implant soft tissue adhesion and stability [9]. Another systematic review of 21 in vitro studies confirmed that abutment surface modifications influence early fibroblast responses [10]. Therefore, clinically relevant three-dimensional (3D) human oral mucosa models are needed to comprehensively evaluate differential attachment of epithelial and connective tissue layers to implant and abutment surfaces under controlled inflammatory conditions.

To simulate the clinical situation as closely as possible, 3D tissue-engineered models of human oral mucosa have been developed for biological evaluation of dental materials, as well as for the investigation of soft tissue attachment to dental implants [11]. Recent applications include inflamed 3D models assessing implant soft tissue attachment via novel pull-testing [12]. The first 3D tissue-engineered transmucosal model was developed by Chai et al. [13] for qualitative and quantitative assessment of mucosal attachment and biological sealing at the peri-implant soft tissue interface [14]. Advanced models now bioengineer junctional epithelium to study epithelial sealing mechanisms [15]. Subsequently, several in vitro studies have utilized reconstructed human oral mucosa with and without oral bacterial components to assess the biological response of oral mucosa to different dental implant and abutment surfaces [16,17,18].

In a very recent in vitro study [12], we investigated the attachment of the soft tissue to different metal, polymer, and ceramic implant surfaces using an inflamed 3D full-thickness tissue-engineered model of human oral mucosa with multiple-endpoint biological analyses, including histology, tissue viability pull-test, and ultrastructural examination of the implant/tissue interface.

Although previous studies provided valuable information regarding the nature of attachment of human oral mucosa as a whole to different implant and abutment surfaces, there is lack of data on differential attachment of oral epithelium and connective tissue layers to different implant surfaces and attachment of distinct layers of human oral mucosa to dental implant and abutment surfaces has not been thoroughly investigated using clinically relevant 3D tissue models.

Emerging bone-mucosa composite models further validate 3D platforms for peri-implant interfaces [19]. Therefore, this study aimed to compare the differential attachment of tissue-engineered oral epithelium, connective tissue layer, and full-thickness human oral mucosa to different metal (TiZr-SLA, TiZr-M), ceramic (ZrO_2_-M, ZrO_2_-P), polymer (PEEK-M), and commercial healing (Ti, ZrO_2_) abutment surfaces using advanced 3D oral mucosal models under simulated inflammatory conditions. These models enable sensitive detection of subtle material-tissue interactions that monolayer cultures and animal studies often overlook, providing novel insights into optimizing peri-implant soft tissue integration for early attachment optimization and potentially long-term clinical success.

## 2. Materials and Methods

### 2.1. Cell Sources and Culture Conditions

Primary human gingival fibroblasts (HGFs) were obtained from the Biorepository at the University of Sheffield (Sheffield, UK). These cells originated from the gingival tissues of healthy donors undergoing routine oral surgical procedures, with written informed consent and ethical approval from the National Research Ethics Service Committee, UK (Reference No. 15/LO/0116). The immortalized human oral keratinocyte cell line OKF6/TERT-2 was kindly provided by the Harvard Institute of Medicine (Boston, MA, USA). The human monocytic THP-1 cell line, derived from acute monocytic leukemia, was obtained from Culture Collections, Public Health England (Porton Down, Salisbury, UK; SP4 0JG).

All cell culture reagents were purchased from Sigma-Aldrich (Dorset, UK), unless otherwise specified. HGFs were cultured in Dulbecco’s Modified Eagle Medium (DMEM; Sigma-Aldrich) supplemented with 10% fetal calf serum (FCS; Gibco, Thermo Fisher Scientific, Waltham, MA, USA), 2 mM L-glutamine (Sigma-Aldrich), and penicillin-streptomycin (100 IU/mL and 100 µg/mL, respectively; Sigma-Aldrich). Oral keratinocytes were maintained in Green’s medium, prepared as described previously by our research group [12].

THP-1 cells were cultured in Roswell Park Memorial Institute medium (RPMI-1640; Sigma-Aldrich) supplemented with 10% FCS (Gibco, Thermo Fisher Scientific) and 2 mM L-glutamine (Sigma-Aldrich).

### 2.2. Test Materials

The materials evaluated in this study were kindly supplied by Institut Straumann AG (Basel, Switzerland) and included the following:

- Gamma-sterilized, sandblasted, and acid-etched titanium–zirconium alloy rods (TiZr-SLA).
- Gamma-sterilized, machined titanium–zirconium alloy rods (TiZr-M).
- Ethylene oxide–sterilized, machined zirconia rods (ZrO_2_-M).
- Ethylene oxide–sterilized, polished zirconia rods (ZrO_2_-P).
- Steam-sterilized, machined polyether ether ketone rods (PEEK-M). PEEK rods were evaluated in their machined state (PEEK-M), reflecting the as-supplied finish and aligning with a previous pilot study in which surface roughness parameters for the same PEEK-M rods were reported. This approach allowed direct comparison across studies and provided insight into the soft tissue response to PEEK under a relatively rough topographical condition [20,21].
- Commercially available sterile titanium healing abutments (HA-Ti) with 4.5 mm height and 5.2 mm diameter.
- Commercially available sterile zirconia healing abutments (HA-ZrO_2_) with 4.5 mm height and 5.2 mm diameter.

Commercial healing abutments exhibit tapered transmucosal profiles, whereas experimental rods maintain parallel cylindrical geometry. This macro-geometric difference represents a study limitation for direct material comparisons, as frictional retention during pull-testing may confound biological attachment measurements. These comparisons, therefore, provide hypothesis-generating observations rather than definitive material rankings.

Surface roughness parameters for TiZr-SLA, TiZr-M, ZrO_2_-M, and PEEK-M rods were previously determined and reported in our earlier pilot study [12].

Additional topographical measurements, including arithmetical mean height (Sa), maximum height (St), and skewness (Ssk), were acquired for Ti and ZrO_2_ healing abutments using a confocal microscope (µSurf Explorer, NanoFocus AG, Oberhausen, Germany) at 20× magnification with a Gaussian filter (cut-off wavelength: 30 µm). Each parameter was recorded from three independent samples, with three measurements per sample.

Given the substantial number of three-dimensional (3D) oral mucosal models required, the investigation was conducted in three experimental phases:

- Stage 1: Full-thickness oral mucosal models were prepared for histological assessment and inflammatory response testing, including TiZr-SLA, TiZr-M, ZrO_2_-M, PEEK-M, and control groups (*n* = 6).
- Stage 2: Split-thickness epithelium–connective tissue models were developed to evaluate differential soft tissue attachment across TiZr-SLA, TiZr-M, ZrO_2_-M, ZrO_2_-P, and PEEK-M groups (*n* = 6).
- Stage 3: Full-thickness oral mucosal models were generated for comparative analysis with commercially available abutments, encompassing all material groups listed above (*n* = 6).

The staged experimental design was strategically implemented to manage construct production constraints while addressing complementary objectives: Stage 1—validated the full-thickness inflamed model and characterized material-specific cytokine responses; Stage 2—isolated differential epithelial vs. connective tissue attachment using split-thickness constructs; and Stage 3—enabled direct comparison of experimental rods versus commercial healing abutments within identical full-thickness models.

### 2.3. 3D Oral Mucosa Models

Three types of three-dimensional (3D) tissue-engineered oral mucosa models were established in this study, as outlined below.

#### 2.3.1. Epithelium-Only Models

Epithelium-only constructs were developed using a multilayer culture of oral keratinocytes grown on an acellular collagen matrix to evaluate epithelial attachment on various implant and abutment surfaces. The acellular matrix was prepared by combining rat-tail type I collagen (5 mg/mL; R&D Systems, Minneapolis, MN, USA) with 10× concentrated DMEM supplemented with 8.5% fetal bovine serum (FBS, *v*/*v*), 2 mM L-glutamine, and a reconstitution buffer composed of 20 mM 4-(2-hydroxyethyl)-1-piperazineethanesulfonic acid (HEPES) and 22 mg/mL sodium bicarbonate. The mixture was maintained on ice and neutralized to pH 7.4 using 1 M sodium hydroxide. A total of 1 mL of the collagen solution was dispensed into tissue culture inserts (0.4 µm pore size; Millipore, Burlington, MA, USA) and incubated at 37 °C for 2 h to allow gelation. The gels were then immersed in complete DMEM for 3 days prior to seeding with keratinocytes (1 × 10^6^ cells per model). Cultures were maintained submerged in Green’s medium for 3 days, then elevated to the air–liquid interface and further incubated for an additional 3 days to promote epithelial stratification before implant placement.

#### 2.3.2. Connective Tissue-Only Models

Connective tissue-equivalent models were designed by embedding oral fibroblasts and THP-1 monocytes within a 3D collagen hydrogel, excluding the epithelial component. These models were employed to evaluate connective tissue responses to different test surfaces. The collagen gel mixture was prepared as described above and maintained on ice. 1 mL of the neutralized gel was combined with a suspension containing 2 × 10^5^ gingival fibroblasts and 1 × 10^5^ THP-1 cells per model. The cell–collagen mixture was dispensed into tissue culture inserts and incubated at 37 °C for 2 h to allow polymerization. The constructs were then completely submerged in complete DMEM and cultured for 3 days before implant placement.

#### 2.3.3. Full-Thickness Oral Mucosal Models

Full-thickness oral mucosal equivalents, composed of stratified oral keratinocytes cultured atop a collagen-based connective tissue populated with gingival fibroblasts and THP-1 monocytes, were employed to assess comprehensive mucosal attachment to different implant and abutment surfaces. The detailed procedures and culture conditions for these full-thickness models have been described previously [12].

### 2.4. Implant and Abutment Insertion

A 4 mm circular section was excised from the center of each 3D tissue model using a biopsy punch. The implant rods and abutments described in Section 2.3 were then positioned in the center of the constructs. Following insertion, the models were maintained in Green’s medium for an additional 3 days to allow further cell–material interaction prior to subsequent analyses.

### 2.5. Induction of Inflammation

To simulate an inflammatory microenvironment, lipopolysaccharide (LPS) derived from *Escherichia coli* (10 µg/mL; Sigma-Aldrich, Dorset, UK) and recombinant tumor necrosis factor-alpha (TNF-α; 25 ng/mL; Sigma-Aldrich, Dorset, UK) were added to the serum-free culture medium at Stage 1 of the experimental protocol. These literature-standard concentrations (LPS 10 μg/mL; TNF-α 25 ng/mL) and 72-h exposure duration follow established 3D oral mucosa protocols that induce reproducible, submaximal inflammatory responses without tissue cytotoxicity, enabling sensitive detection of material-dependent differences [12,22]. The treatment was applied to 3D mucosal models containing different implant materials, including TiZr-SLA, TiZr-M, ZrO_2_-M, and PEEK-M, to induce a controlled inflammatory response.

### 2.6. Multiple Endpoint Analyses

After completion of the culture period, the tissue models were processed for multiple-endpoint analyses as described below:

#### 2.6.1. Tissue Viability Pull Test

A combined mechanical and biological method was employed to assess epithelial and connective tissue adhesion to various implant and abutment surfaces. After 72 h of incubation, the implant rods and abutments were carefully extracted from the center of the 3D tissue constructs. Implant rods and abutments were extracted manually using sterile forceps by a single calibrated operator, with the tissue insert secured in a custom holder. All devices were withdrawn along their longitudinal axis in a single continuous motion at a visually standardised speed, while maintaining perpendicular alignment to the tissue construct to minimise shear forces. The metabolic activity of adherent cells remaining on the implant surfaces was then quantified using the PrestoBlue cell viability reagent (10% *v*/*v* in additive-free DMEM; Thermo Fisher Scientific, Waltham, MA, USA). Each rod was placed in a 48-well plate with 1 mL of the reagent solution and incubated overnight at 37 °C and 5% CO_2_. Triplicate 100 µL aliquots from each well were transferred to a 96-well plate and fluorescence was measured using a plate reader (Infinite 200 PRO; Tecan Trading AG, Männedorf, Switzerland) with excitation at 530 nm and emission at 590 nm.

#### 2.6.2. Histological Processing

The 3D tissue constructs were fixed in 10% (*v*/*v*) neutral buffered formalin for 24 h, followed by implant rod removal and standard paraffin embedding. Sections of 5 µm thickness were cut, mounted on slides, and stained with hematoxylin and eosin (H&E). Slides were independently evaluated by two blinded observers (E.B. and K.M.) under a light microscope (Olympus BX51; Olympus Corporation, Tokyo, Japan), with digital images captured using an integrated camera. Sections from healthy human gingival biopsy tissue served as positive controls.

#### 2.6.3. Scanning Electron Microscopy

Post-pull test, cells retained on implant rod surfaces were examined via scanning electron microscopy (SEM). Specimens were fixed in 10% formalin supplemented with 2% osmium tetroxide, dehydrated through an ascending ethanol series (30–100%), and air-dried overnight. Dried samples were mounted on aluminum stubs, sputter-coated with gold using an Edwards S150B coater, and imaged with a Tescan Vega3 SEM (Tescan, Brno, Czech Republic) at 15 kV accelerating voltage (Electron Microscopy Facility, University of Sheffield, UK).

#### 2.6.4. Measurement of the Release of Pro-Inflammatory Markers

Cell culture supernatants collected from Stage 1 experiments were assayed for pro-inflammatory cytokines, including interleukin (IL)-1β (Sigma-Aldrich, Dorset, UK), IL-6, and IL-8 (R&D Systems, Minneapolis, MN, USA), using commercially available enzyme-linked immunosorbent assay (ELISA) kits following the manufacturers’ protocols. Absorbance was read at 450 nm with wavelength correction at 570 nm on a microplate reader.

#### 2.6.5. Statistical Analysis

A sample size of *n* = 6 per group was selected based on power calculations from our prior 3D oral mucosa studies [12], which demonstrated ≥80% power to detect medium-to-large effect sizes (Cohen’s d ≥ 0.8) in Presto Blue viability and ELISA cytokine assays at α = 0.05, balancing statistical rigor with tissue engineering feasibility. Quantitative results are expressed as mean ± standard deviation (SD) for each experimental group. Differences between groups were analyzed using One-way or Two-way analysis of variance (ANOVA) followed by Tukey’s post-hoc test in GraphPad Prism software version 10. *p* values were determined and values of *p*  <  0.05, *p* < 0.01, *p* < 0.001 (*, **, *** respectively) were considered significant.

## 3. Results

### 3.1. Tissue-Engineered Oral Mucosa Recapitulates Native Gingival Histology with Stratified Epithelium and Viable Fibroblast/THP-1 Connective Tissue

**Figure 1:**
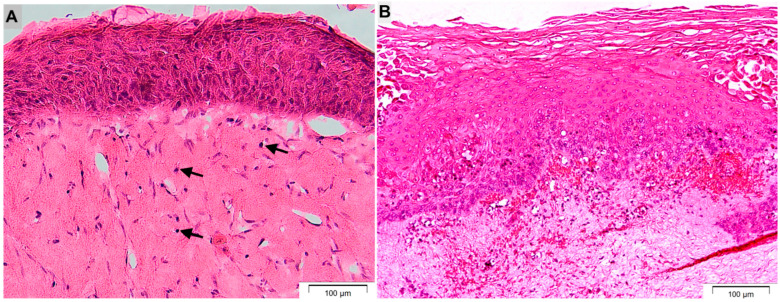
Histological characterization of tissue-engineered oral mucosa compared with native gingival mucosa. Microscopic views of hematoxylin and eosin–stained sections of tissue-engineered oral mucosa (**A**) and normal human gingival mucosa (**B**). The tissue-engineered model shows a well-stratified, multilayered oral epithelium overlying a fibroblast- and THP-1 monocyte–populated connective tissue compartment (arrows), closely resembling the histological architecture of native gingival mucosa. Scale bars: 100 µm.

Microscopic views of the histological sections of tissue-engineered oral mucosa (A) and normal human gingival mucosa (B) are presented in Figure 1. The images show the presence of a stratified and multi-layered oral epithelial layer and an underlying connective tissue with viable fibroblasts and THP-1 monocyte cells in the 3D oral mucosal model, which highly resembles the histological appearance of the native gingival mucosa.

### 3.2. TiZr-SLA Surfaces Promote Superior Epithelial and Connective Tissue Attachment Compared with Machined and Polished Alternatives

**Figure 2:**
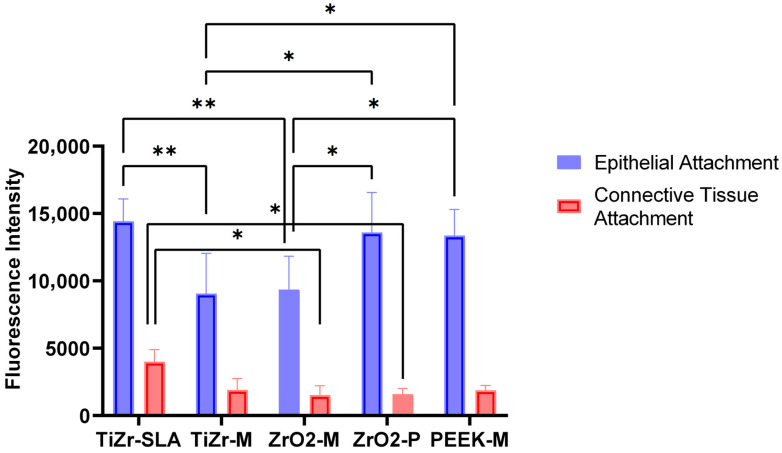
Differential epithelial and connective tissue attachment to experimental implant surfaces. PrestoBlue fluorescence intensity representing epithelial attachment and connective tissue attachment to TiZr-SLA, TiZr-M, ZrO-M, ZrO-P, and PEEK-M rods after 72 h of culture, expressed as remaining viable cells on the implant surface following the pull test (*n* = 6). TiZr-SLA exhibited significantly higher connective tissue attachment compared to ZrO-M (*p* = 0.0489) and ZrO-P, while epithelial attachment was greater on TiZr-SLA, ZrO-P, and PEEK-M compared to TiZr-M and ZrO-M. Statistical analysis was performed using Two-way ANOVA with Tukey’s post hoc test; * *p* < 0.05, and ** *p* < 0.01.

The results of the PrestoBlue tissue viability assay for the epithelial and connective tissue attachment tests are presented in Figure 2. Statistical analysis of the data revealed greater epithelial attachment to TiZr-SLA, ZrO_2_-P, and PEEK-M surfaces (*p* < 0.05) compared to ZrO_2_-M and TiZr-M. Notably, the TiZr-SLA surface exhibited a statistically significantly higher connective tissue attachment level compared to the other groups, particularly ZrO_2_-M (*p* = 0.0489) and ZrO_2_-P (*p* = 0.027).

### 3.3. Commercial Titanium and Zirconia Healing Abutments Exhibit Highest Overall Soft Tissue Viability Post-Pull Test Versus Experimental Rods

**Figure 3:**
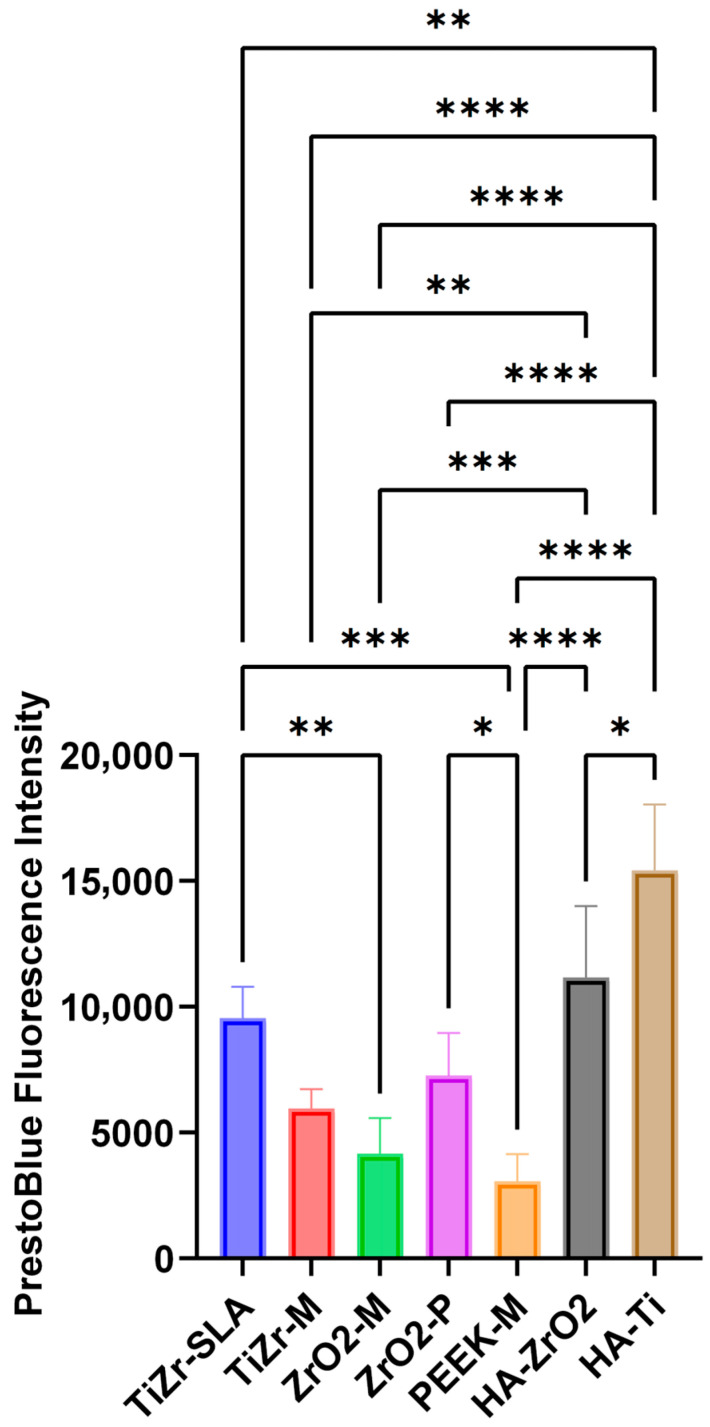
Overall soft tissue attachment of full-thickness 3D oral mucosal models to experimental rods and healing abutments. PrestoBlue fluorescence intensity (mean ± SD) indicating total viable soft tissue remaining attached to TiZr-SLA, TiZr-M, ZrO_2_-M, ZrO_2_-P, PEEK rods, and Ti and ZrO_2_ healing abutments after the pull test performed on full-thickness oral mucosal models. Commercial healing abutments and selected experimental surfaces demonstrated higher overall soft tissue attachment compared with other materials. Statistical differences were evaluated using one-way ANOVA with Tukey’s post hoc test. * *p* < 0.05, ** *p* < 0.01, *** *p* < 0.001, **** *p* < 0.0001.

Figure 3 demonstrates the results of the PrestoBlue assay for overall soft tissue attachment of the full-thickness oral mucosal models to different rods and healing abutments. Statistical analysis showed the commercially available ZrO_2_ and Ti healing abutments, TiZr-SLA, and ZrO_2_-P experimental rods had the highest remaining soft tissue viability after the implant pull test (*p* < 0.05). There was also a statistically significant difference in tissue viability between TiZr-M, ZrO_2_-M, and PEEK-M groups (*p* < 0.05), and the viability decreased respectively.

### 3.4. SEM Reveals Extensive Cell Retention on Rough TiZr-SLA Surfaces with Patchier Coverage on Machined TiZr-M and PEEK-M Implants

**Figure 4:**
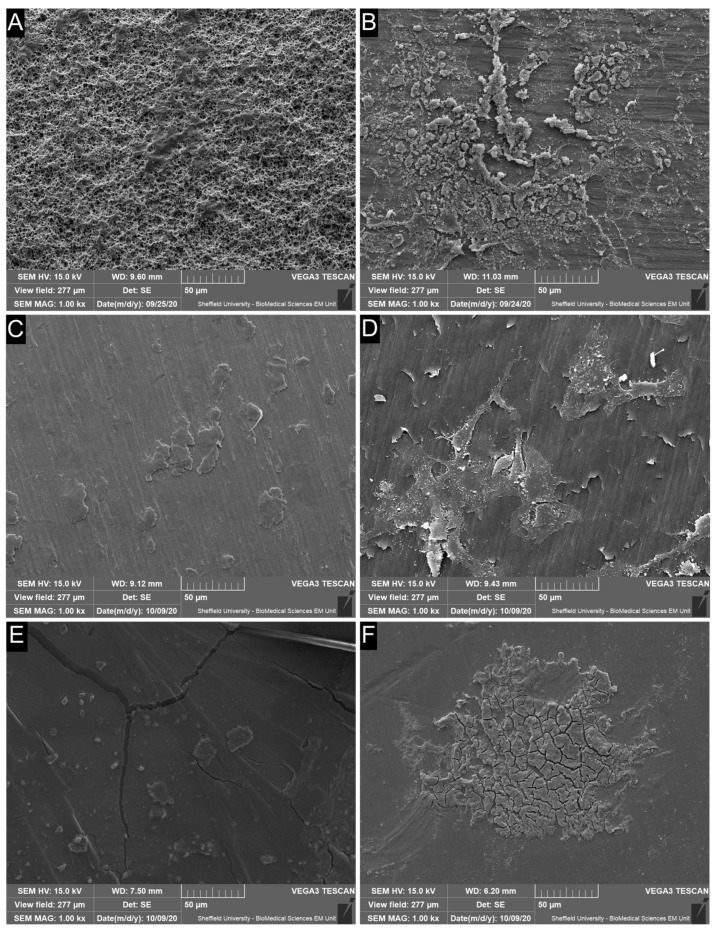
Scanning electron microscopy (SEM) of soft tissue attachment on different implant surfaces after pull testing. Representative SEM images showing the surface topography and residual soft tissue cells on (**A**,**B**) rough TiZr-SLA, (**C**,**D**) machined TiZr-M, and (**E**,**F**) PEEK-M rods following the tissue viability pull test. TiZr-SLA exhibits a highly micro-roughened surface with extensive cell coverage, whereas TiZr-M and PEEK-M present smoother surfaces with more limited and patchy cell remnants. All images acquired at 1000× magnification; scale bars: 50 µm.

The scanning electron microscopy (SEM) micrographs of the oral mucosa tissue, which remained attached to different implant surfaces after the pull test, are presented in Figure 4. The images display distinct characteristics of TiZr-SLA, TiZr-M, ZrO_2_-M, and PEEK-M surfaces and the morphology of the cells attached onto different surfaces. TiZr-SLA rods had a roughened surface structure, while TiZr-M, ZrO_2_-M, and PEEK-M rods had smoother surfaces with some grooves that were acquired during the manufacturing process. All surfaces showed evidence of cell attachment. The mean and standard deviation (SD) values for Sa, St, and Ssk of the surface topographies of ZrO_2_ healing abutments were 0.021 (0.001) µm, 0.157 (0.01) µm, and −0.241 (0.053), respectively.

The corresponding values for pure Ti healing abutments were 0.248 (0.058) µm, 1.540 (0.446) µm, and 0.506 (0.205) µm, respectively.

### 3.5. ZrO-M and PEEK-M Implant Surfaces Induce Significantly Higher IL-1β Release, Promoting Pro-Inflammatory Responses in 3D Oral Mucosal Models

**Figure 5:**
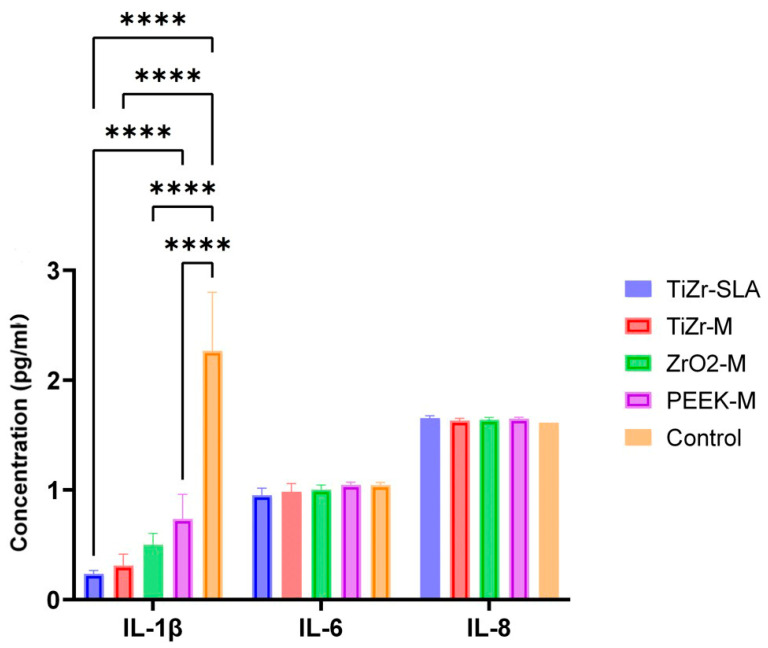
Differential Pro-Inflammatory Cytokine Secretion from 3D Oral Mucosal Models on Experimental Implant Surfaces. IL-1β, IL-6, and IL-8 concentrations (pg/mL) in culture supernatants after 72 h exposure to TiZr-SLA, TiZr-M, ZrO-M, ZrO-P, and PEEK-M rods, measured by ELISA (450 nm). ZrO-M and PEEK-M exhibited significantly elevated IL-1β levels as compared to other implants. 3D oral mucosal models cultured without implant punching served as controls. The other exposure parameters remained the same in the control group. Analysis was performed using one-way ANOVA with Tukey’s post-hoc test. Results are representative of six independent experiments, and bar graphs display average ± SD. **** *p* < 0.0001.

ELISA analysis of the cell culture supernatants for the presence of pro-inflammatory cytokines revealed that the ZrO_2_-M and PEEK-M groups had statistically significantly higher levels of IL-1β released from the oral mucosal models compared to TiZr-SLA, TiZr-M, and ZrO-P (*p* < 0.0001; Figure 5). In contrast, IL-6 and IL-8 concentrations showed no significant differences across all tested surfaces, with ZrO-P exhibiting moderately higher but non-significant levels. Hence, only IL-1β differed significantly between materials.

## 4. Discussion

Although osseointegration has been studied for many years, the interaction of oral soft-tissues with dental implant and abutment surfaces has received little attention. It has been primarily reported in conjunction with research findings on hard-tissue interfaces [23,24,25]. Attachment of both the gingival epithelium and the connective tissue layers to the implant and abutment is an important factor in maintaining early peri-implant stability, mucosal health, and preventing apical ingress of oral bacterial biofilms [26]. The epithelial components include oral epithelium, peri-implant sulcular epithelium, and a thin peri-implant epithelium, which is attached to the implant surface via hemidesmosomes [27,28]. The underlying connective tissue layer includes functionally oriented collagen fibres, abundant fibroblasts, and some limited vascular components due to the absence of the periodontal ligament [29,30,31]. The attachment of the connective tissue layer to the implant surface is reported to be achieved by a circumferential seal via dense collagen fibres surrounding the implant, without penetrating collagen fibres, in contrast to natural teeth [3,32,33]. Therefore, the soft tissue biological seal around implants is believed to be weaker and more vulnerable to bacterial invasion than that of the natural teeth, as achieved by the dento-gingival attachment [29,31].

Different materials, such as metals (pure Ti and its alloys), ZrO_2_ (ceramics), polymers (PEEK), and carbons, have been used to fabricate the transmucosal components of implants and abutments [1]. Various surface treatment and modification methods have been employed to enhance the hard and soft tissue attachment to the implant surfaces. These include sand-blasting, acid etching, calcium-phosphate coating, laser treatment, electrochemical anodic oxidation, and photofunctionalization [34].

There are conflicting reports in implantology literature on whether the implant material type has a significant influence on the soft tissue attachment. For instance, some studies have demonstrated the presence of soft-tissue hemidesmosomes and optimal junctional epithelium formation on Ti, ZrO_2,_ and aluminum-based ceramic surfaces, but poor soft tissue attachment to gold and dental ceramics [28,35,36]. Welander et al. [37] showed a reduced number of fibroblasts, inflammatory cells, and peri-implant collagen fibres around gold abutments compared to Ti and ZrO_2_ abutments. On the contrary, other studies have reported that abutment materials had no effects on the dimensions of peri-implant soft tissues [38,39,40].

The results of our experiments indicated that the type of abutment material and its surface characteristics can significantly affect the differential attachment of the oral epithelial and connective tissue layers, as assessed by the 3D tissue-engineered oral mucosal models. SLA TiZr surface showed significantly higher levels of both epithelial and connective tissue attachment compared to TiZr-M surface. These findings are consistent with a previous in vitro study [41] which suggested that the behaviour of human gingival fibroblasts is influenced by the implant surface wettability and topography. In addition, a recent systematic review of 21 in vitro studies indicated that abutment material and its mechanical, physical, and chemical modification influence fibroblast response, especially in the earlier phases of contact with the abutment surface [10]. It is important to note that most of the previous in vitro studies were based on 2D monolayer cell culture systems, and this is the first study that has used a 3D tissue-engineered oral mucosal model to investigate the differential attachment of oral epithelial and connective tissue layers to different implant and abutment surfaces.

Our results also indicate 3D models detected material-specific differences (*p* = 0.0489 TiZr-SLA vs. ZrO_2_-M) with higher statistical power (*n* = 6 vs. typical animal *n* = 3–4) than many published animal studies which often fail to reach significance due to low sample sizes. Cochran et al. [30] demonstrated that SLA and Ti plasma-sprayed (TPS) implant surfaces had similar supra-crestal soft tissue dimensions. Schwarz et al. [42] suggested that hydrophilic SLA Ti surfaces may have the potential to enhance connective tissue attachment to the implant. Linares et al. [43] investigated the soft tissue histomorphology at the transmucosal part of machined and modified implants and reported the percentage of connective tissue attached to the implant surface. The length of connective tissue attachment was longer at the modified TiZr surface compared to Ti-M implants, although the soft tissue interface was similar among all test groups.

In our study, ZrO_2_-P and PEEK-M surfaces exhibited comparable epithelial attachment, both significantly higher than machined ZrO_2_-M (*p* < 0.05). However, connective tissue attachment was similar across ZrO_2_-P, ZrO_2_-M, and PEEK-M surfaces. Notably, while clinical PEEK abutments are typically finished to a highly polished surface to minimize plaque retention [44,45], our machined PEEK-M represents an inherently rougher topography that is expected to amplify inflammatory responses, compared to production-polished clinical components. This machining approach aligns with our prior pilot characterization of surface roughness parameters and provides valuable insight into soft tissue responses under suboptimal topographical conditions [12]. Future studies should systematically compare machined versus polished PEEK within identical 3D models to elucidate surface finishing effects on epithelial and connective tissue integration. These findings align with Nothdurft et al. [46], who reported complex, differential adhesion behaviors of gingival fibroblasts and epithelial cells to ZrO_2_ and Ti alloy surfaces across machined, polished, and SLA topographies in monolayer culture. Their study concluded that the response of fibroblasts and epithelial cells was influenced by both the type of implant material and its surface topography. A review of the literature, including three human studies, three animal studies, and six in vitro studies, indicated that epithelial cells appear to slightly favour ZrO_2_ and polished Ti surfaces [47].

Commercially available Ti and ZrO_2_ healing abutments showed the highest level of overall soft tissue attachment compared to the experimental implant rods in this study. Although this could be due to different surface chemistry, processing conditions, and the formation of an oxide layer on the abutment surface, it could also be attributed to the differences between the contour of the healing abutments, which had a tapered design, and the shape of the experimental rods, which had parallel walls. The influence of abutment shape and contour on soft tissue attachment has not been thoroughly investigated.

The greater soft tissue viability observed on commercial Ti and ZrO_2_ healing abutments must be interpreted with caution, as these components have a tapered emergence profile, whereas the experimental rods were strictly cylindrical with parallel walls. The pull test captures combined effects of biological adhesion and mechanical friction; therefore, undercuts and taper may enhance frictional resistance independently of true cell–surface attachment. This macro-geometric mismatch can be a confounder and precludes definitive material-based comparisons between commercial abutments and experimental rods in this study. Further research using clinically relevant 3D in vitro tissue models is required to establish the influence of abutment shape and emergence profile on early attachment of peri-implant oral epithelial and connective tissue layers.

As previously mentioned, several recent in vitro studies have used tissue-engineered oral mucosa for the biological evaluation of dental implants and abutments [16,17,18]. One of the major advantages of our 3D oral mucosal model was that it was able to detect the small differences in early soft tissue attachment of different layers of the oral mucosa to different abutment surfaces with a high level of sensitivity. The implant pull test was an innovative modification in this study that enabled measurement of soft tissue attachment and produced quantitative data that could be analysed statistically.

SEM analysis enabled visualization of the cells remaining attached to different implant surfaces after the pull test. Morphology of the attached cells on implant surfaces has been discussed thoroughly in our previous report [12]. The epithelial compartment utilized OKF6/TERT-2 immortalized keratinocytes with primary gingival fibroblasts (HGFs) and THP-1 monocytes, creating a hybrid model that balances reproducibility with physiological relevance. OKF6/TERT-2 forms stratified epithelia histologically identical to primary cells, with proper hemidesmosome formation and cytokine profiles on implant surfaces. Paired with primary HGFs, this hybrid detected significant material-specific attachment differences (TiZr-SLA, ZrO-P, PEEK-M > TiZr-M, ZrO-M; *p* < 0.05), demonstrating superior sensitivity versus primary-only models hampered by donor variability. Multi-endpoint validation confirms OKF6/TERT-2 recapitulates key peri-implant functions, establishing this hybrid as a robust translational platform [12,48,49]. The nature of cell attachment onto Ti implant surfaces with hemidesmosomes has been demonstrated by Chai et al. [28] using transmission electron microscopy analysis of resin-embedded ultra-thin sections of implant-soft tissue interface using tissue-engineered oral mucosal models.

Regarding the release of pro-inflammatory cytokines, ZrO_2_-M and PEEK-M groups showed higher levels of IL-1β, a potent upstream mediator that amplifies NF-κB activation and recruits innate immune cells [50], as detected by ELISA, and compared to the other groups. However, there was no statistically significant difference in the amount of IL-6 and IL-8 released from the oral mucosal models among the different groups tested in this study. The cytokines tested in this study (specifically IL-1β) are the most clinically relevant cytokines for oral biocompatibility evaluation of biomaterials, as they have been directly associated with gingivitis and periodontal diseases [51]. In a clinical human study, Degidi et al. [52] examined the gingival biopsies obtained from around Ti and ZrO_2_ healing abutments after six months of implant placement and reported higher levels of inflammatory cell infiltrate at Ti sites compared to ZrO_2_. Sanz-Sánchez, Sanz-Martín, Carrillo de Albornoz, Figuero and Sanz [53] also observed more pronounced inflammatory responses and increased bleeding on probing around Ti abutments compared to ZrO_2_. The differences in observations between our in vitro study and the abovementioned clinical studies could be due to the presence of oral bacteria in the clinical situation, which has been shown to colonise at higher numbers on Ti surfaces than ZrO_2_ [54,55] and may significantly influence the inflammatory response of peri-implant soft tissues. Further studies are required to investigate the inflammatory response of tissue-engineered human gingival mucosa to different types of abutments in the presence of oral bacterial biofilms. The pronounced IL-1β upregulation observed specifically with machined zirconia (ZrO-M) and PEEK-M surfaces after 72 h, with IL-6 and IL-8 remaining comparable across all groups, as seen in Figure 5, underscores a material- and topography-dependent activation of the NLRP3 inflammasome in gingival fibroblasts and macrophages within our 3D model. IL-1β, as a primary upstream cytokine, that can be released rapidly via inflammasome activation in macrophage- and fibroblast-rich 3D mucosal constructs without necessarily triggering a sustained IL-6/IL-8 response under simplified in vitro conditions, triggers pyroptotic cell death and amplifies NF-κB signaling, which may disrupt hemidesmosome formation and collagen fiber orientation critical for the peri-implant seal [56,57], hence correlating directly with the reduced connective tissue attachment observed on these surfaces, as suggested by our data (Figure 2, Figure 3 and Figure 4). This selective IL-1β response, without concomitant IL-6/IL-8 elevation, suggests an acute, non-chronic inflammatory profile, potentially driven by zirconia’s different surface characteristics and chemistry and PEEK’s hydrophobic polymer nature, which impede protein adsorption compared to hydrophilic SLA titanium [58]. These findings align with clinical observations of elevated peri-implant inflammation around non-Ti ceramics, yet diverge by highlighting early IL-1β as a predictive biomarker for long-term soft tissue stability, superior to downstream chemokines, in preclinical implant evaluation [59]. Future studies incorporating LPS-challenged co-cultures could validate this inflammasome axis, informing preclinical material optimization toward cytokine-modulating surface chemistries.

A key limitation is the difference in macro-geometry between experimental rods and commercial healing abutments. The tapered contour of the abutments may have increased frictional retention during extraction, thereby overestimating soft tissue attachment relative to parallel-walled rods. Another limitation of the pull test is the manual extraction of implants, which provides a semi-quantitative assessment of the pull rate and force; future studies should incorporate mechanical testing systems to standardize these parameters. In addition, the 3D in vitro model used in this study does not contain the vascular components of the native human oral mucosa and further studies on development of vascularized tissue engineered human oral mucosa models would be beneficial to further simulate the clinical situation in assessment of implant/soft tissue interactions.

## 5. Conclusions

This study demonstrates superior soft tissue attachment to commercial healing abutments which could be potentially geometry-influenced, highlighting the 3D model’s value for preclinical optimization of transmucosal implant surfaces to minimize plaque retention and improve soft tissue attachment. TiZr-SLA conferred maximal connective tissue adhesion and polished zirconia and machined PEEK favored epithelial attachment. Only IL-1β showed significant material-specific inflammatory responses. These findings establish 3D human oral mucosa models as useful platforms for in vitro evaluation of soft tissue interactions with transmucosal implant surfaces.

## Data Availability

The original contributions presented in this study are included in the article. Further inquiries can be directed to the corresponding author.
